# Osthole Attenuates Hepatic Injury in a Rodent Model of Trauma-Hemorrhage

**DOI:** 10.1371/journal.pone.0065916

**Published:** 2013-06-06

**Authors:** Huang-Ping Yu, Fu-Chao Liu, Yung-Fong Tsai, Tsong-Long Hwang

**Affiliations:** 1 Department of Anesthesiology, Chang Gung Memorial Hospital, Taoyuan, Taiwan; 2 College of Medicine, Chang Gung University, Taoyuan, Taiwan; 3 Graduate Institute of Clinical Medical Sciences, Chang Gung University, Taoyuan, Taiwan; 4 Graduate Institute of Natural Products, Chang Gung University, Taoyuan, Taiwan; Van Andel Institute, United States of America

## Abstract

Recent evidences show that osthole possesses anti-inflammatory properties and protective effects following shock-like states, but the mechanism of these effects remains unknown. The p38 mitogen-activated protein kinase (p38 MAPK) pathway exerts anti-inflammatory effects in injury. The aim of this study was to investigate whether p38 MAPK plays any role in the osthole-mediated attenuation of hepatic injury after trauma-hemorrhage. Male Sprague-Dawley rats underwent trauma-hemorrhage (mean blood pressure maintained at approximately 35–40 mmHg for 90 minutes), followed by fluid resuscitation. During resuscitation, a single dose of osthole (3 mg/kg, intravenously) with and without a p38 MAPK inhibitor SB-203580 (2 mg/kg, intravenously), SB-203580 or vehicle was administered. Plasma alanine aminotransferase (ALT) with aspartate aminotransferase (AST) concentrations and various hepatic parameters were measured (n = 8 rats/group) at 24 hours after resuscitation. The results showed that trauma-hemorrhage increased hepatic myeloperoxidase activity, intercellular adhesion molecule-1 and interleukin-6 levels, and plasma ALT and AST concentrations. These parameters were significantly improved in the osthole-treated rats subjected to trauma-hemorrhage. Osthole treatment also increased hepatic phospho-p38 MAPK expression compared with vehicle-treated trauma-hemorrhaged rats. Co-administration of SB-203580 with osthole abolished the osthole-induced beneficial effects on the above parameters and hepatic injury. These results suggest that the protective effect of osthole administration on alleviation of hepatic injury after trauma-hemorrhage, which is, at least in part, through p38 MAPK-dependent pathway.

## Introduction

Liver injury following trauma-hemorrhage can result in serious life threatening conditions [1], [2]. Studies have shown that trauma-hemorrhage can induce massive pro-inflammatory mediators production and subsequent accumulation of neutrophils in the liver [3]. Neutrophils are activated following trauma-hemorrhage and can release superoxide anions and proteolytic enzymes [3]–[5]. Intercellular adhesion molecule (ICAM)-1 is enhances a firm adhesion of neutrophils to the vascular endothelium, and markedly up-regulated following trauma-hemorrhage [6], [7]. Interleukin-6 (IL-6) plays a significant role in neutrophil infiltration and hepatic inflammation following organ injury [3], [8]. Furthermore, there is convincing evidence that IL-6 is required for the expression of adhesion molecules [9].

The p38 mitogen-activated protein kinase (p38 MAPK) pathway affects pro-inflammatory cytokines production and chemotactic events in response to injury [6]. In addition, the p38 MAPK pathway has a pivotal role in neutrophils migration to undergo chemotaxis [10]. P38 MAPK also plays an important role in shock-induced hepatic, myocardial and intestinal injuries [10]–[12]. Previous studies have also shown that up-regulation of the p38 MAPK pathway attenuates the overproduction of cytokines, adhesion molecules, and neutrophil accumulation after trauma-hemorrhage [6], [10], [12].

Osthole can protect against organ injury following shock-like states [13]. Previous studies have also shown that osthole can reduce cytokine production, and attenuate lipopolysaccharide-induced acute lung injury [14]. Furthermore, previous studies have shown that an increase in p38 MAPK activity improves liver function following trauma-hemorrhage or ischemia injury [10]. It is implied that p38 MAPK may play a role in osthole-mediated hepatoprotection following trauma-hemorrhage. We hypothesized that the beneficial effects of osthole following trauma-hemorrhage are mediated via a p38 MAPK-related pathway. To test this hypothesis, animals were treated with osthole alone and in combination with the p38 MAPK inhibitor SB-203580 after trauma-hemorrhage. The effects of these treatments were then examined with respect to hepatic injury as well as hepatic myeloperoxidase (MPO) activity, ICAM-1, IL-6, and phospho (p)-p38 MAPK/p38 MAPK levels following trauma-hemorrhage.

## Materials and Methods

### Animals

Adult male Sprague-Dawley strain rats were used in this study. The rats were obtained from the National Science Council Experimental Animal Center. All animal experiments were performed according to the guidelines of the *Animal Welfare Act* and *The Guide for Care and Use of Laboratory Animals* from the National Institutes of Health. All procedures and protocols were approved by the Institutional Animal Care and Use Committee of Chang Gung Memorial Hospital.

### Rat Trauma-Hemorrhage Model

A non-heparinized rat model of trauma-hemorrhage was used in this study [15]. Thirty-six male Sprague-Dawley rats (275–325 g) were randomly assigned to 6 groups (n = 6/group). Initial studies examined trauma-hemorrhage, with the groups receiving osthole (0, 0.3, 1, 3, or 10 mg/kg); sham groups were also included. In addition, forty-eight male Sprague-Dawley rats were randomly divided into 6 separate groups (n = 8/group). All animals were placed in the animal house individually in cages with air-conditioned (humidity 70–75%), controlled temperature (24–25°C) and lighting (light– dark cycle every 12 hours: lights on 06:00 to 18:00). Basal diet and water was provided and allowed at least 1 week to adapt to the environment. Before initiation of the experiment, male Sprague- Dawley rats were fasted overnight but allowed free water access. Trauma-hemorrhage and resuscitation was then performed as described previously [20]. In brief, rats were anesthetized by isoflurane inhalation, and a 5-cm midline laparotomy was performed to induce soft tissue trauma. The abdominal wound was then closed in layers. Polyethylene catheters (PE-50; Becton Dickinson & Co., Sparks, MD) were placed in both femoral arteries and the right femoral vein from bilateral inguinal incision wounds (about 0.5 cm in length), and the bilateral inguinal incision sites were then closed. The wounds were bathed with 1% lidocaine (Elkins-Sinn Inc., Cherry Hill, NJ) throughout the operative procedure to reduce postoperative pain. The rats were allowed to awaken, after which they were bled rapidly within 10 minutes to a mean arterial pressure of 35 to 40 mmHg. This level of hypotension was maintained until the animals could no longer maintain a mean arterial pressure of 40 mmHg unless some fluid in the form of Ringer's lactate was administered. This time was defined as maximum bleed-out. After the maximal bleed-out, mean arterial pressure was maintained between 35 to 40 mmHg until 40% of the maximal bleed-out volume was returned in the form of Ringer's lactate solution (about 90 minutes from the onset of bleeding). The rats were then resuscitated with four times the volume of the shed blood with Ringer's lactate for 60 minutes. Thirty minutes before the end of the resuscitation period, the rats received osthole (3 mg/kg, intravenously), osthole plus the p38 MAPK inhibitor SB-203580 (2 mg/kg, intravenously at the beginning of resuscitation), SB-203580, or an equal volume of the vehicle (about 0.3 mL, DMSO). After resuscitation, the catheters were removed, the vessels ligated, and the skin incisions closed with sutures. Sham-operated animals underwent all operative procedures, but neither hemorrhage nor resuscitation was performed. Vehicle or osthole was also administered in sham-operated rats after catheters were placed. The animals were humanely killed at 24 hours after the end of resuscitation or sham operation. In the experiment under review, there were 8 rats in each group.

### Measurement of Hepatic Injury

At 24 hours after trauma-hemorrhage or sham operation, blood samples with heparin were obtained and plasma was separated by centrifugation. Hepatic injury was determined by measuring plasma levels of AST and ALT using a colorimetric analyzer (Dri-Chem 3000; Fuji Photo Film Co., Tokyo, Japan).

### Measurement of MPO Activity

MPO activity in homogenates of liver tissues was determined as described previously [15]. Frozen tissue samples were thawed and suspended in phosphate buffer (pH 6.0) containing 0.5% hexadecyltrimethylammonium bromide (Sigma, St. Louis, MO). The samples were sonicated on ice, centrifuged at 12,000 g for 15 minutes at 4°C, and an aliquot was transferred into phosphate buffer (pH 6.0) containing 0.167 mg/mL o-dianisidine hydrochloride and 0.0005% hydrogen peroxide (Sigma, St. Louis, MO). The change in absorbance at 460 nm was measured spectrophotometrically for 5 minutes. MPO activity was calculated using a standard curve that was generated using human MPO (Sigma, St. Louis, MO), and values were normalized to protein concentration.

### Measurement of ICAM-1 and IL-6 Levels

The liver tissues were homogenized in PBS (1∶10 weight:volume; pH 7.4) containing protease inhibitors (Complete Protease Inhibitor Cocktail; Boehringer, Mannheim, Germany). The homogenates were centrifuged at 2,000 g for 20 minutes at 4°C and the supernatant was analyzed for the presence of ICAM-1 and IL-6 using ELISA kits (R&D, Minneapolis, MN) according to the manufacturer's instructions and as described previously [15]. An aliquot of the supernatant was used to determine protein concentration by the Bio-Rad DC Protein Assay (Bio-Rad, Hercules, CA).

### Western Blot Assay

Rat liver tissues were homogenized in a buffer as described previously [6]. The homogenates were centrifuged at 12,000 g for 15 minutes at 4°C, analyzed using SDS-PAGE, and the proteins were then transferred to nitrocellulose membranes. The membranes were incubated with antibodies for p38 MAPK protein, p-p38 MAPK (Cell Signaling Technology, Beverly, MA), or GAPDH (Abcam, Cambridge, MA) overnight at 4°C. The membranes were incubated with horseradish peroxidase-conjugated goat anti-rabbit antibody or goat anti-mouse antibody for 1.5 hours at room temperature. After the final washing, blots were probed using enhanced chemiluminescence (Amersham, Piscataway, NJ) and autoradiographed.

### Statistical Analysis

For statistical analysis we used the InStat 3.0 biostatistics program (Graph Pad Software Inc., San Diego, CA). Results are presented as mean ± standard error of the mean (SEM). The data were analyzed using one-way analysis of variance (ANOVA) and the Tukey test, and differences were considered significant at *p*≤0.05.

## Results

### Dose-Response Effects of Osthole on Plasma AST and ALT Levels

**Figure 1:**
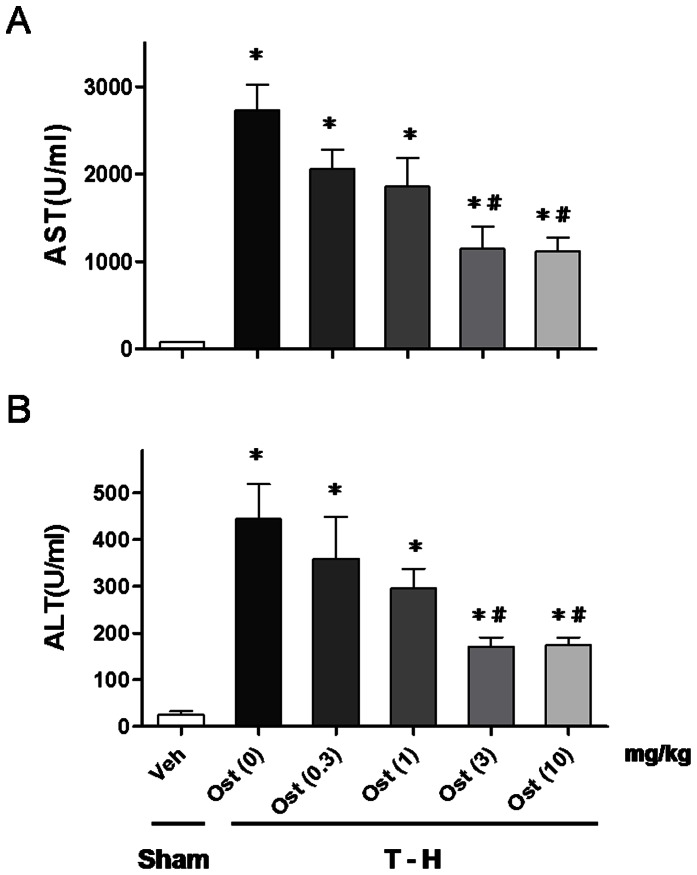
Dose-dependent responses to osthole treatment of plasma AST (A) and ALT (B) in rats at 24 hours after sham operation (sham) or trauma-hemorrhage and resuscitation (T-H). Animals were treated with osthole (Ost) at doses of 0, 0.3, 1, 3, or 10 mg/kg. Data are shown as the mean ± SEM. n = 6 rats in each group. ^*^ *p*<0.05 compared with sham; ^#^ *p*<0.05 compared with T-H + Ost (0 mg/kg).

As shown in Figures 1A and 1B, trauma-hemorrhage was related to a significant increase in plasma AST and ALT levels at 24 h after resuscitation (AST: 2732.0±291.5 *vs*. 81.5±3.0; ALT: 444.2±74.8 *vs*. 25.8±6.6 U/ml, *p*<0.05). Administration of osthole at a dose of 0.3, 1, 3, or 10 mg/kg was used to evaluate the effects of osthole on the attenuation of hepatic injury after trauma-hemorrhage. As shown in Figure 1, there was a diminished benefit when osthole was administered at the dose of 0.3 or 1 mg/kg [AST: 2058.0±216.4 (0.3 mg/kg), 1855.0±334.7 (1 mg/kg); ALT: 359.0±89.5 (0.3 mg/kg), 295.8.0±41.6 (1 mg/kg) U/ml]. The effects of osthole were equivalent when administered at a dose of 3 or 10 mg/kg [AST: 1142.0±255.5 (3 mg/kg), 1118.0±157.7 (10 mg/kg); ALT: 171.3±19.6 (3 mg/kg), 174.8±16.1 (10 mg/kg) U/ml].

### Alteration in Plasma AST and ALT Levels

**Figure 2:**
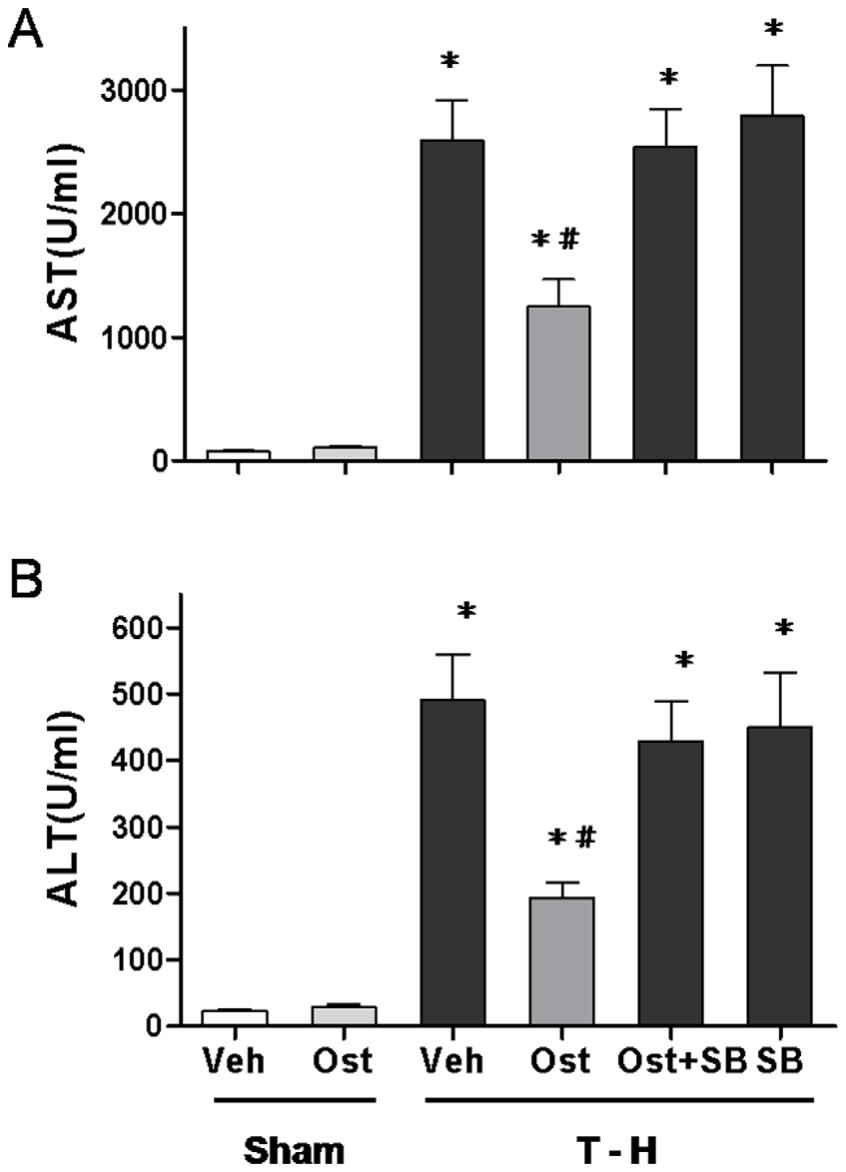
Effect of osthole treatment on plasma AST (A) and ALT (B) in rats at 24 hours after sham operation (Sham) or trauma-hemorrhage and resuscitation (T-H). Animals were treated with either vehicle (Veh), osthole (Ost), osthole in combination with SB-203580 (Ost+SB) or SB-203580 (SB). Data are shown as mean ± SEM of 8 rats in each group. ^*^ *p*<0.05 compared to Sham; ^#^ *p*<0.05 compared to T-H+Veh, T-H+Ost+SB and T-H+SB.

As shown in Figures 2A and 2B, no significant difference in plasma AST and ALT levels was observed between vehicle- and osthole-treated sham groups (AST: 79.0±6.7 *vs*. 109.7±12.5; ALT: 23.2±2.5 *vs*. 28.8±4.2 U/ml). At 24 hours after trauma-hemorrhage, there were significant increases in plasma AST and ALT levels. Osthole (3 mg/kg) treatment attenuated the trauma-hemorrhage-induced increase in plasma AST and ALT levels (AST: 1248.0±221.3 *vs*. 2593.0±321.6; ALT: 193.7±22.8 *vs*. 490.2±68.9 U/ml, *p*<0.05). To determine whether the salutary effects of osthole in attenuating hepatic injury after trauma-hemorrhage were mediated via a p38 MAPK-mediated activity, a group of osthole-treated trauma-hemorrhage rats were administrated with the p38 MAPK inhibitor SB-203580. The results indicated that administration of the p38 MAPK inhibitor SB-203580 prevented the osthole-induced decrease in plasma AST and ALT levels [AST: 1248.0±221.3 (osthole) *vs*. 2541.0±297.3 (osthole + SB-203580); ALT: 193.7±22.8 (osthole) *vs*. 428.8±60.4.1 (osthole + SB-203580) U/ml, *p*<0.05].

### Alteration in Hepatic MPO Activity

**Figure 3:**
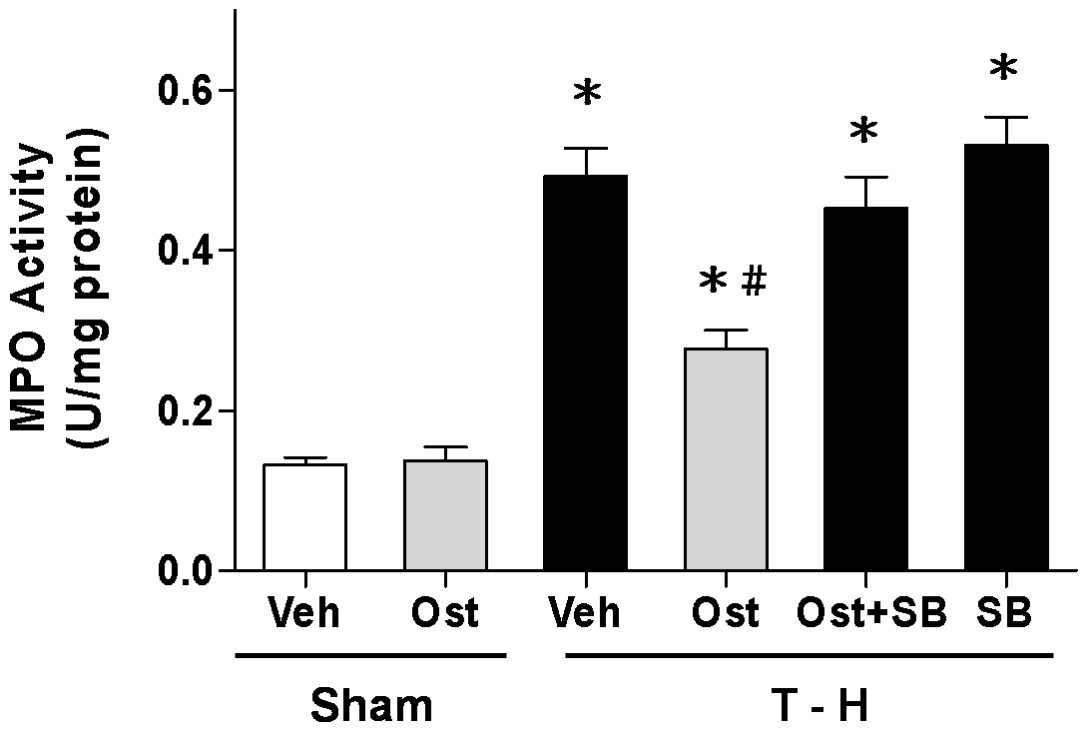
Effect of osthole treatment on hepatic MPO activity in rats at 24 hours after sham operation (Sham) or trauma-hemorrhage and resuscitation (T-H). Animals were treated with either vehicle (Veh), osthole (Ost), osthole in combination with SB-203580 (Ost+SB) or SB-203580 (SB). Data are shown as mean ± SEM of 8 rats in each group. ^*^ *p*<0.05 compared to Sham; ^#^ *p*<0.05 compared to T-H+Veh, T-H+Ost+SB, and T-H+SB.

Hepatic MPO activity in sham or trauma-hemorrhaged animals, with and without osthole treatment, was shown in Figure 3. In sham-operated rats, osthole did not alter hepatic MPO activity (0.133±0.009 *vs*. 0.138±0.017 U/mg protein). Trauma-hemorrhage resulted in a significant increase in hepatic MPO activity in vehicle-treated animals (0.493±0.035 *vs*. 0.133±0.009 U/mg protein, *p*<0.05). Osthole treatment attenuated the increase in hepatic MPO activity (0.277±0.023 *vs*. 0.493±0.035 U/mg protein, *p*<0.05). Furthermore, administration of the p38 MAPK inhibitor SB-203580 prevented the osthole-mediated attenuation of hepatic MPO activity after trauma-hemorrhage [0.453±0.039 (osthole + SB-203580) *vs*. 0.277±0.023 (osthole) U/mg protein, *p*<0.05].

### Alteration in Hepatic ICAM-1 Concentrations

**Figure 4:**
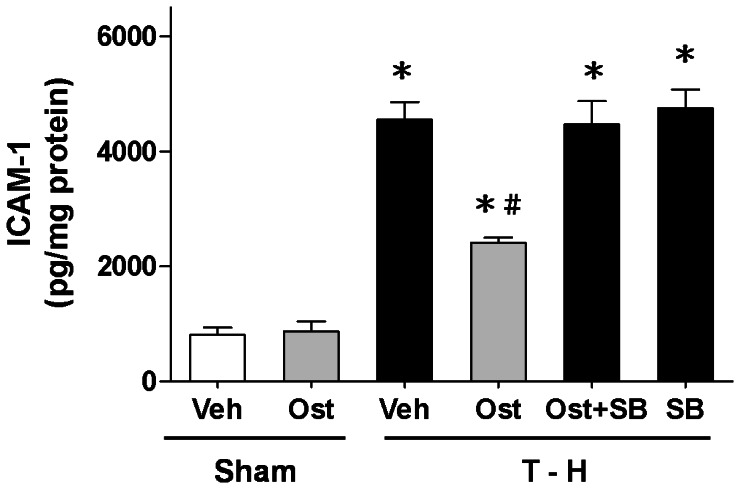
ICAM-1 levels in the liver in rats after sham operation (Sham) or trauma-hemorrhage and resuscitation (T-H). Animals were treated with vehicle (Veh), osthole (Ost), osthole in combination with SB-203580 (Ost+SB) or SB-203580 (SB). Data are shown as mean ± SEM of 8 rats in each group. ^*^ *p*<0.05 compared to Sham; ^#^ *p*<0.05 compared to T-H+Veh, T-H+Ost+SB, and T-H+SB.

Trauma-hemorrhage significantly increased ICAM-1 concentrations in the liver (4546.0±312.5 *vs*. 807.5±133.2 pg/mg protein, *p*<0.05) (Figure 4). Treatment with osthole attenuated the trauma-hemorrhage-induced increase in ICAM-1 concentrations (2410.0±87.6 *vs*. 4546.0±312.5 pg/mg protein, *p*<0.05). Co-administration of the p38 MAPK inhibitor SB-203580 with osthole prevented the osthole-induced reduction in ICAM-1 concentrations [4465.0±408.4 (osthole + SB-203580) *vs*. 2410.0±87.6 (osthole) pg/mg protein, *p*<0.05].

### Alteration in Hepatic IL-6 Levels

**Figure 5:**
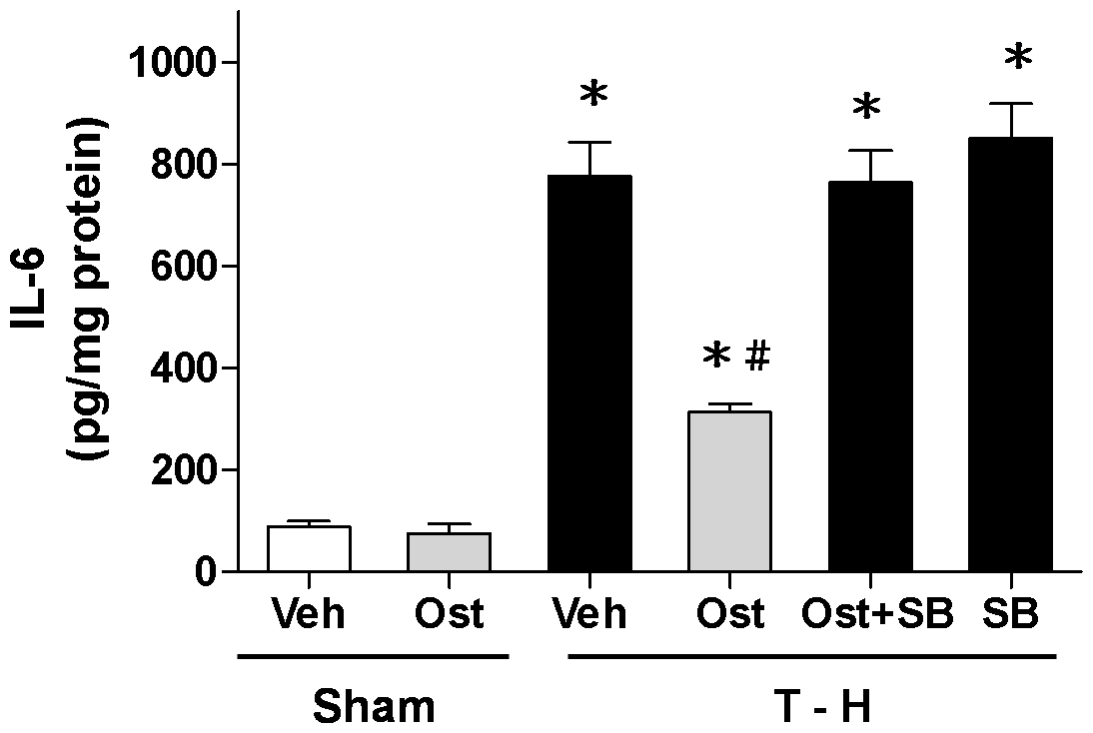
Effect of osthole treatment on hepatic IL-6 levels in rats at 24 hours after sham operation (Sham) or trauma-hemorrhage and resuscitation (T-H). Animals were treated with either vehicle (Veh), osthole (Ost), osthole in combination with SB-203580 (Ost+SB) or SB-203580 (SB). Data are shown as mean ± SEM of 6 rats in each group. ^*^ *p*<0.05 compared to Sham; ^#^ *p*<0.05 compared to T-H+Veh, T-H+Ost+SB, and T-H+SB.

There was no significant difference in hepatic IL-6 levels between the vehicle- and osthole-treated sham groups (Figure 5). Trauma-hemorrhage significantly increased hepatic IL-6 levels in vehicle-treated rats compared with sham animals (776.9±66.1 vs. 89.1±10.1 pg/mg protein, *p*<0.05). The increase in hepatic IL-6 levels was reduced by osthole treatment, and the osthole-mediated reduction in IL-6 levels was abolished by p38 MAPK inhibitor SB-203580 co-administration [313.3±16.1 (osthole) *vs*. 764.1±61.8 (osthole + SB-203580) pg/mg protein, *p*<0.05].

### Hepatic P38 MAPK Protein Expression and Activity

**Figure 6:**
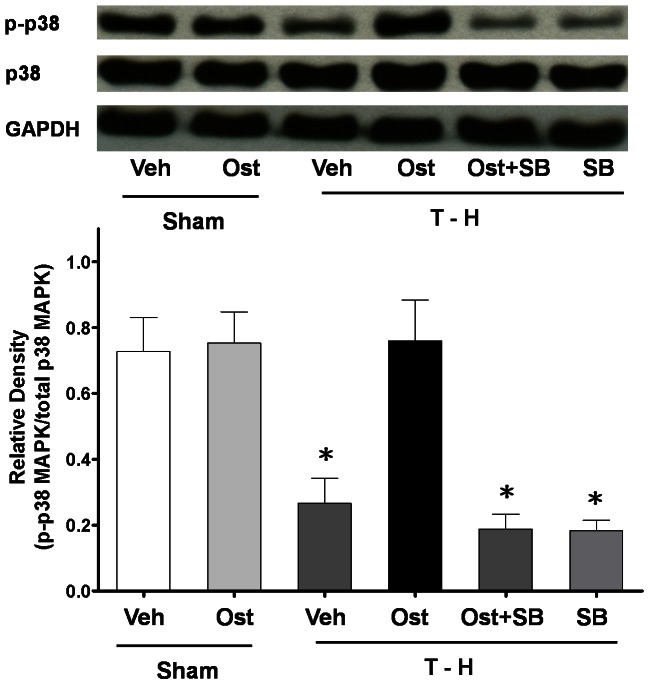
Hepatic p-p38 MAPK and p38 MAPK protein expressions from sham-operated animals receiving vehicle (Sham+Veh; lane 1) or osthole (Sham+Ost; lane 2), trauma-hemorrhage animals receiving vehicle (T-H+Veh; lane 3), osthole (T-H+Ost; lane 4), osthole and SB-203580 (T-H+Ost+SB; lane 5) or SB-203580 (T-H+SB; lane 6). Blots were reprobed for GAPDH as a control for equal protein loading in all lanes. The bands were analyzed using densitometry, and the values are presented as mean ± SEM for 8 rats in each group. ^*^ *p*<0.05 versus all other groups.

There was no significant difference in p38 MAPK protein expression between the sham and trauma-hemorrhaged rats (Figure 6). However, p38 MAPK activity as determined by its phosphorylation was significantly decreased after trauma-hemorrhage (0.27±0.08 *vs*. 0.73±0.10, *p*<0.05). Administration of osthole after trauma-hemorrhage restored p38 MAPK activity to the levels observed in the sham animals (0.76±0.12 *vs*. 0.73±0.10). The increase in phosphorylated-p38 MAPK induced by osthole was abolished when SB-203580 was administered along with osthole [0.19±0.05 (osthole + SB-203580) *vs*. 0.76±0.12 (osthole), *p*<0.05] (Figure 6).

## Discussion

In this study, we sought to determine whether p38 MAPK-dependent pathways play an important role in osthole-mediated hepatoprotection following trauma-hemorrhage. The salutary effects of osthole at doses of 3 mg/kg have been evaluated in hepatic injury after trauma-hemorrhage. Our results indicated that administration of osthole attenuated trauma-hemorrhage-induced hepatic injury. In addition, 24 hours after trauma-hemorrhage, hepatic MPO activity, ICAM-1 and IL-6 levels were markedly increased in male rats. Administration of osthole (3 mg/kg) during resuscitation attenuated the increases in those parameters. Administration of osthole also prevented the trauma-hemorrhage-induced decrease in p-p38 MAPK expression. Furthermore, our findings indicated that administration of the p38 MAPK inhibitor SB-203580 along with osthole abolished the osthole-induced hepatoprotection in rats subjected to trauma-hemorrhage. These studies collectively suggest that the salutary effects of osthole seem to be mediated via a p38 MAPK-related pathway.

The liver is considered to be a critical organ in patients suffering from traumatic trauma-hemorrhagic injuries that can lead to the development of multiple organ failure [2], [16]. Previous studies have shown that hepatic injury is associated with increased neutrophil accumulation [15], [17]. The infiltration of neutrophils in the liver is also accompanied by increased expression of cytokines and adhesion molecules [15], [18]. Tissue MPO activity is an indicator of neutrophil infiltration, and it has been correlated with ICAM-1 expression after trauma-hemorrhage [6], [7]. Our results showed that trauma-hemorrhage resulted in a significant increase in hepatic ICAM-1 levels, which was accompanied by elevated hepatic MPO activity. However, ICAM-1 levels and MPO activity were attenuated in osthole-treated trauma-hemorrhaged rats. In addition, IL-6 is an important pro-inflammatory mediator in hepatic damage and is required for adhesion molecule expression [19]. Liver injury or hypoxia causes marked increases in hepatic IL-6 expression [7], [20], [21]. In this study, hepatic IL-6 levels were significantly attenuated in the animals treated with osthole after trauma-hemorrhage.

Osthole is reported to reduce cytokine production and tissue injury following shock-like states [13], [14], [22]. Osthole administration inhibits focal inflammation reaction, and prevents brain against ischemic damage by reducing cytokines, and cyclooxygenase-2 release [22]. Osthole treatment also attenuates ischemic stroke via inhibition of MPO activity and pro-inflammatory cytokines production in a rat model of middle cerebral artery occlusion [23]. Previous studies have also shown that osthole significantly diminishes tumor necrosis factor-α and cyclooxygenase-2 expressions and nuclear factor-κB activity in lipopolysaccharide-stimulated macrophages [24]. The ability of osthole to mediate expression of inflammatory cytokines suggests a role for osthole in the regulation of liver inflammation. Taken together, osthole might reduce inflammation through down-regulation of pro-inflammatory mediators.

The p38 MAPK pathway is known to play an important role in the cell survival and organ protection through p38 MAPK phosphorylation [6]. Studies have shown that activation of the p38 MAPK pathway protects organs or cells against ischemia-reperfusion injury [25]. Inhibition of the p38 MAPK pathway with the p38 MAPK inhibitor SB-203580 increases inducible nitric oxide synthase expression and high-mobility group box 1 levels, and decreases the survival of mice subjected to sepsis [26]. Our previous studies have also reported that p38 MAPK pathway plays a critical role in organ protection [6], [27]. Our finding showed that trauma-hemorrhage was accompanied by a decrease in hepatic p38 MAPK activation. The depressed p38 MAPK phosphorylation following trauma-hemorrhage was restored by administration of osthole after trauma-hemorrhage. However, the increase in p38 MAPK phosphorylation by osthole after trauma-hemorrhage was abolished by co-administration of SB-203580. These results thus indicate that the salutary effects of osthole on hepatic function after trauma-hemorrhage are in part mediated by a p38 MAPK-dependent pathway.

Previous studies have shown that osthole attenuates renal and intestinal ischemia- reperfusion injury that is associated with decreased neutrophil infiltration, oxidative stress, caspase-3, and nitric oxide levels [13], [28]. Previous reports also indicate that pretreatment with osthole decreases traumatic brain injury by suppressing oxidative stress and cell apoptosis [29]. There are other mechanisms that may possibly be responsible for the favorable effect offered by osthole. The finding that osthole can attenuate hepatic dysfunction and inflammatory responses when administered therapeutically, i.e. during the resuscitation phase, may be important.These data may have translational significance and clinical relevance. Our findings have also provided insights into the mechanism by which osthole therapeutically attenuates hepatic dysfunction and inflammatory responses after trauma-hemorrhage.

In conclusion, our study indicates that osthole administration ameliorates hepatic injury and production of pro-inflammatory mediators after trauma-hemorrhage. Blockade of p38 MAPK activation abolishes the salutary effects of osthole in the liver following trauma-hemorrhage. Our findings provide evidence that osthole-mediated hepatoprotection is, in part, mediated via a p38 MAPK-dependent pathway after trauma hemorrhage. Osthole may be a novel adjunct for improving depressed hepatic function under adverse circulatory conditions.
